# Selection and Validation of Reliable Reference Genes for Gene Expression Studies in Different Genotypes and TRV-Infected Fruits of Peach (*Prunus persica* L. Batsch) during Ripening

**DOI:** 10.3390/genes13010160

**Published:** 2022-01-17

**Authors:** Ze Xu, Jieyu Dai, Weijing Su, Haixia Wu, Kamran Shah, Libo Xing, Juanjuan Ma, Dong Zhang, Caiping Zhao

**Affiliations:** College of Horticulture, Northwest Agriculture and Forestry University, Yangling, Xianyang 712100, China; xuzeizi@163.com (Z.X.); jieyu204@163.com (J.D.); swj10162021@163.com (W.S.); zetianwu2021@163.com (H.W.); kamranshah801@nwafu.edu.cn (K.S.); libo_xing@nwsuaf.edu.cn (L.X.); mjj@nwafu.edu.cn (J.M.); afant@nwafu.edu.cn (D.Z.)

**Keywords:** data normalization, different genotypes, *Prunus persica*, reference gene, TRV-infected fruits

## Abstract

Real-time quantitative PCR (RT-qPCR) is a powerful tool to detect and quantify transcription abundance, and the stability of the reference gene determines its success. However, the most suitable reference gene for different genotypes and tobacco rattle virus (TRV) infected fruits was unclear in peach (*Prunus persica* L. Batsch). In this study, 10 reference genes were selected and gene expression was characterized by RT-qPCR across all samples, including different genotypes and TRV-infected fruits during ripening. Four statistical algorithms (geNorm, NormFinder, BestKeeper, and RefFinder) were used to calculate the stability of 10 reference genes. The geNorm analysis indicated that two suitable reference genes should be used for gene expression normalization. In general, the best combination of reference genes was *CYP2* and *Tua5* for TRV-infected fruits and *CYP2* and *Tub1* for different genotypes. In *18S*, *GADPH,* and *TEF2*, there is an unacceptable variability of gene expression in all experimental conditions. Furthermore, to confirm the validity of the reference genes, the expression levels of *PpACO1*, *PpEIN2*, and *PpPL* were normalized at different fruit storage periods. In summary, our results provide guidelines for selecting reliable reference genes in different genotypes and TRV-infected fruits and lay the foundation for accurate evaluation of gene expression for RT-qPCR analysis in peach.

## 1. Introduction

Gene expression analysis is becoming increasingly important for understanding the signaling and metabolic pathways during cell development and death [1,2]. Real-time quantitative PCR (RT-qPCR) is one of the most widely used techniques to determine gene expression because of its good repeatability, strong specificity, high sensitivity, and wide application range compared with microarrays, semi-quantitative, and northern blots [3,4,5]. In RT-qPCR, using a reference gene to normalize gene expression is a simple, popular method with wide application [6]. The reference gene, also known as the housekeeping gene, is stably expressed in various tissues and experimental conditions and maintains the minimal life function of cells [7,8]. Normalization can lead to incorrect results if there are large fluctuations in the expression of reference genes [6]. Consequently, selecting an optimal reference gene with a steady expression pattern is an essential pre-requisite before normalization.

Many studies have reported the selection and validation of reference genes in plants [9,10,11,12,13,14,15]. However, these reference genes exhibit constant expression patterns under certain experimental conditions, but their expression levels vary widely under other experimental conditions. In longan, *CYP2* was the best reference gene at the fruit development stages, but the least stable gene under NAA treatment [16]. In mulberry, *TIP41* was the preferred reference gene for normalizing gene expression under control, but not under drought stress [17]. In the case of peach, *CYP2* was identified as a stable reference gene in fruit flesh samples, but displayed unacceptably variable expression patterns in fruit peel [11]. Therefore, it is completely necessary to select stably expressed reference genes under specific experimental conditions to normalize the target gene, rather than using a reference gene previously published under other experimental conditions.

Peach (*Prunus persica* L. Batsch) is one of the most economically important fruit crops in China. Softening is a complex process of biochemistry and physiology, including cell wall destruction, pectin hydrolysis, and content release [18]. According to the texture characteristics, peaches are classified into melting flesh (MF), non-melting flesh (NMF), and stony-hard (SH) types [19,20]. Significant differences exist in the softening rates of peach fruits with different textures [21]. MF peach is rapidly softening, SH peach is hardly softened on the tree and after harvest, and NMF peach is between MF and SH [22,23]. The study of the softening of peach fruits with different genotypes is an important part of the postharvest problem. Therefore, clarifying the expression patterns of key regulatory factors during fruit ripening can help to better elucidate the complex regulatory mechanisms.

Virus-induced gene silencing (VIGS) is one of the most widely used and efficient tools for gene function research [24,25,26]. VIGS technology requires a virus vector to achieve silencing of endogenous genes. Tobacco rattle virus (TRV) vector has been successfully used in most fruit trees. Bai et al. had succeeded in knocking down the *PpCCD4* gene with the TRV-VIGS approach in white flesh peaches [27]. In Li et al. study [28], *PpSEP1* expression was inhibited by TRV-VIGS to determine *PpSEP1* functions in peach during ripening. Thus, VIGS is the most important molecular method to verify gene function in peach. Finding the most suitable internal reference gene in TRV-infected fruits is an indispensable and important part of the RT-qPCR experiment. Although Kou et al. found the best reference genes under different tissue and different exogenous regulator treatments in peach [11], the selected reference genes may not be suitable for TRV-injected peach fruits.

Reference genes are generally stable expressed genes that maintain minimal vital functions of the cell, such as ubiquitin (*UBQ*) and RNA polymerase subunit (*RP*) [7]. In the current study, actin protein, tublin and 18S ribosomal RNA (*18S*) are the most commonly used as internal reference genes for gene expression analysis in peach [13,15,28]. Many stably expressed reference genes reported in other species, such as glyceraldehyde-3-phosphate dehydrogenase (*GADPH*), translation enlongation factor (*TEF*), and cyclophilin (*CYP*), were also included as candidate genes for subsequent analysis [14,17]. In this study, 10 reference genes were selected according to reference genes often used and reported in peach. Their expression level in peach was confirmed by RT-qPCR across all samples, including different genotypes and TRV-infected fruits. The stability of the expression of 10 reference genes was analyzed by geNorm, NormFinder, BestKeeper, and RefFinder. In addition, the expression profiles of *PpACO1*, *PpEIN2*, and *PpPL* were normalized by using the unstable reference genes and the best combination of reference genes, respectively. Our data provides validated reference genes for RT-qPCR analysis of peach fruit, which will be conducive to future studies on the gene expression of peach.

## 2. Materials and Methods

### 2.1. Different Genetypes Fruits

Peach (*P. persica* L. Batsch) varieties ‘Rui Hong’ (RH, melting flesh), ‘Zao Feng Wang’ (ZFW, melting flesh), ‘Babygold 5’ (B5, non-melting flesh), and ‘Qin Wang’ (QW, stony-hard) were used as experimental materials and planted in the Experimental Station of the College of Horticulture at Northwest A&F University, Yangling, Shaanxi, China. During the commercial harvest period, the fruit maintained relatively high hardness, the green surface of the fruit faded slightly, and some skin turned red. The fruits were harvested at the same maturity with a uniform size and no mechanical damage. The harvested fruit was soaked for 30 min in 2 g/L of carbendazim to kill worm eggs, and then stored at 24 ± 1 °C. During storage, fruits were sampled every two days, frozen in liquid nitrogen, and stored at −80 °C until the fruit was completely softened. Three biological repetitions were designed, and each biological repetition was performed by mixing the pulp of five fruits. Table S1 lists the sample data used for this experiment.

### 2.2. TRV-Infected Fruits

The fruits of the peach varieties ‘Rui Hong’ and ‘Zao Feng Wang’ are used as TRV-injected fruits. The empty plasmid pTRV1 and pTRV2 were transformed into Agrobacterium strain GV3101 by heat stimulation and grown on LB medium containing kanamycin and rifampicin at 28 °C for 3 days. A single clone was picked and grown in LB medium containing kanamycin and rifampicin for 14 h, and then the cells were collected and resuspended to an OD600 of 0.8 and stored at room temperature for 2 h as described by Bai et al. [27]. According to the protocols reported by Li et al. [28], 200 intact RH fruits were selected randomly for injection. The culture containing the recombinant plasmids pTRV1 and pTRV2 was mixed at a ratio of 1:1, and then 200 μL of the culture was infiltrated into the peach fruit with a needle. One week after the injection, the fruits were harvested and stored at 24 ± 1 °C. During storage, fruits were sampled every two days, frozen in liquid nitrogen, and stored at −80 °C until the fruit was completely softened. Three biological repetitions were designed, and each biological repetition was performed by mixing the pulp of five fruits.

### 2.3. Design for RT-qPCR Primers

Primers of ten reference genes (*18S*, *Actin*, *CYP2*, *TEF2*, *GAPDH*, *PLA2*, *RPII*, *Tua5*, *Tub1*, and *UBQ10*) had previously been designed in other studies [9,11,29,30,31,32], and the details are listed in Table S2. The mRNA sequences of *PpACO1*, *PpEIN2* and *PpPL* were obtained from the peach genome database on the GDR website (https://www.rosaceae.org/, accessed on 15 July 2021). The primer was designed by Primer Premier 6, and the specificity was tested by electrophoresis in a 1.0% agarose gel. Primer sequence of *PpACO1*, *PpEIN2*, and *PpPL* was listed in Table S3.

### 2.4. RNA Isolation and cDNA Preparation

Total RNA was extracted using the RNAprep Pure Plant Kit (Polysaccharides & Polyphenolics-rich) (Tiangen Biotech, Beijing, China). RNA quality and integrity were tested by electrophoresis in 1% agarose gels and a microspectrophotometer (Thermo NanoDrop 2000, Wilmington, NC, USA), respectively. Only RNA preparations having an A260/A280 value of 1.8–2.0 and an A260/A230 value of >2.0 were used for subsequent analysis. Reverse transcription was completed using the PrimeScript RT Reagent Kit with gDNA Eraser (Takara, Beijing, China).

### 2.5. RT-qPCR with SYBR Green

RT-qPCR was performed with the Bio-Rad CFX system (Bio-Rad Laboratories, Hercules, CA, USA). Each reaction mix contains 2 µL of ddH_2_O, 1 µL of forward and reverse primer mixture, 1 µL cDNA, and 5 µL of SYBR Premix ExTaq II (TaKaRa, Beijing, China) to a final volume of 10 µL. The PCR amplification procedure was set to 95 °C for 1 min, followed by 40 cycles of 95 °C for 15 s, 60 °C for 20 s, and 72 °C for 15 s, and constructed a melting curve. Each RT-qPCR reaction was carried out in triplicate. A no-template control (NTC) was also carried out.

### 2.6. Normalization of PpACO1, PpEIN2, and PpPL

*PpACO1* (ethylene synthesis-related gene), *PpEIN2* (ethylene signal transduction-related gene), and *PpPL* (cell wall degradation-related gene) all play important roles in peach ripening and have high expression levels during storage [18,28]. In this study, to validate the reference genes, the expression levels of the three selected genes were normalized using the best reference gene combination, the best reference gene and the unstable gene in peach varieties ‘Rui Hong’ (RH), ‘Qin Wang’ (QW), and TRV-infected ‘Zao Feng Wang’ (ZFW) fruit during storage. The expression level of the three genes was calculated by the 2-Δ Δ Ct method [33]. 

### 2.7. Data Analysis

The geNorm algorithm program was used by qbase + software (Demo 3.2) [34]. The Normfinder [35] and BestKeeper [36] algorithm programs were Excel-based tools to calculate the stability of 10 reference genes. RefFinder [37] is an online website (https://www.heartcure.com.au/reffinder/, accessed on 27 July 2021) to carry out comprehensive stability analysis. 

## 3. Result

### 3.1. Detection of Amplification Specificity of Reference Gene Primers

Based on previous studies [9,11,29,30,31,32], we selected 10 candidate reference genes to re-evaluate gene expression stability in TRV-based VIGS-injected peach fruits in order to identify the most suitable reference gene for RT-qPCR. The housekeeping genes were those genes involved in encoding 18S ribosomal RNA (*18S*), actin protein (*Actin*), cyclophilin 2 (*CYP2*), translation elongation factor 2 (*TEF2*), glyceraldehyde-3-phosphate dehydrogenase (*GADPH*), phospholipase A2 β (*PLA2*), RNA polymerase subunit (*RPII*), tublin α-5 (*Tua5*), tublin β-1 (*Tub1*), and ubiquitin 10 (*UBQ10*). All the RNA samples were detected by electrophoresis in 1% agarose gels, and the 28S rRNA and 18S rRNA bands were clear. The result of electrophoresis showed a specific single band for all RT-qPCR products (Figure S1), and the melting curve figures displayed a single peak for each RT-qPCR product (Figure S2), demonstrating that the primers for the 10 reference genes used in this study have high specificity.

### 3.2. Expression Level of Reference Genes 

**Figure 1:**
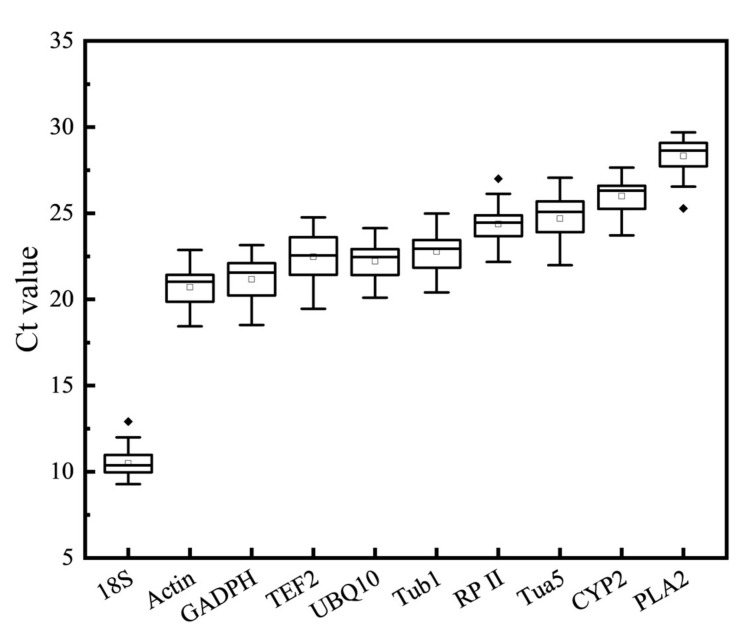
Real-time quantitative PCR (RT-qPCR) raw Ct values for ten reference genes in all experimental samples. The boxes represent the 25th and 75th percentiles. A line and a square in each box indicate the median and mean Ct values, respectively. The black dots represent potential outliers.

In order to further confirm the raw expression levels, a RT-qPCR was designed for the 10 reference genes for all samples, including different genotypes and TRV-infected fruits. The Ct values of the 10 reference genes under all experimental conditions range from 9.28 for *18S* to 29.69 for *PLA2*, which indicates that obvious differences exist in the expression profile (Figure 1). The average Ct value of *18S* is the lowest, indicating that it has the lowest number of cycles to reach threshold fluorescence and it exhibits the highest expression abundance. Except for *18S*, *CYP2*, and *PLA2*, the mean Ct values of other genes varied between 20 and 25, demonstrating that those genes were relatively highly expressed in this study. The least expressed genes were *CYP2* and *PLA2*, with Ct values of 26.03 and 28.37, respectively. Furthermore, *18S* had a narrowest Ct range compared to other genes, while *TEF2* had the widest, and the majority of the reference genes had lower expression variation. 

### 3.3. Stability of Reference Genes

In our study, the stability of 10 reference genes were independently confirmed using the geNorm, NormFinder, BestKeeper, and RefFinder across all samples, including different genetypes and TRV-infected fruits.

#### 3.3.1. geNorm Analysis

**Figure 2:**
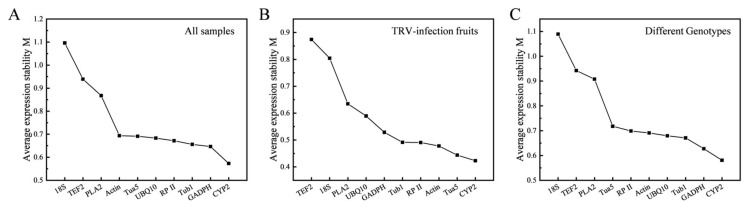
The measurement (M) values of ten reference genes were calculated by geNorm and ranked across all the samples (**A**), TRV-infected fruits (**B**), and different genotypes (**C**). A higher M value means lower stability, while a lower M value implies more stable gene expression.

The geNorm was used to calculate the measurement (M) values of expression stability for 10 reference genes based on the raw Ct value. As shown in Figure 2, reference genes were ranked according to M values. A higher M value means lower stability, while a lower M value implies more stable gene expression. In our study, 10 reference genes had M values below 1.5, which is a threshold for assessing stability, indicating that all genes had a stable expression profile across all these experimental sets. In the total sample, the most stably expressed genes were *CYP2* and *GADPH*, and the two least stably expressed genes were *18S* and *TEF2*. The result was similar across the different genotypes. In the TRV-infected fruits, *CYP2* and *Tua5* were the most stably expressed genes, whereas *18S* and *TEF2* were the least.

**Figure 3:**
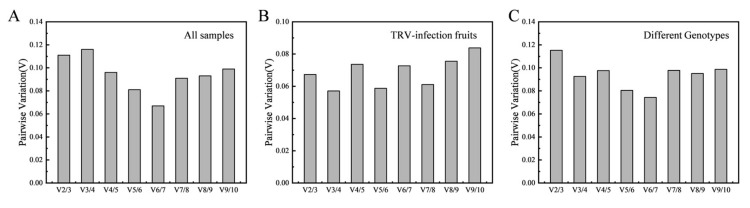
The pairwise variation (Vn/Vn + 1) of ten reference genes by geNorm in all the samples (**A**), TRV-infected fruits (**B**), and different genotypes (**C**). Cut-off value is 0.15.

The appropriate number of reference genes was also evaluated based on the pairwise variation value (Vn/n + 1 value) (Figure 3). The result showed that the V2/3 of all experimental groups was lower than 0.15, indicating that two suitable reference genes were required for normalization. The optimal combination of reference genes was *CYP2* with *Tua5* for TRV-infected fruits and *CYP2* with *GADPH* for different genotypes. In addition, *CYP2* with *GADPH* was selected as the best reference gene combination in all samples.

#### 3.3.2. NormFinder Analysis

The expression stability was further analyzed by NormFinder, in which intra- and inter-group were considered to calculate normalization, and ANOVA was used to evaluate expression stability. Unlike qbase + software, NormFinder cannot directly use the raw data, which needs to be transformed into quantities for relative comparison. The variation value for 10 reference genes were calculated by NormFinder, and a higher stable value meant lower stability, whereas lower values indicated better stability. In this sense, all samples with no subgroup and the two series were calculated. The result of the variation values is shown in Table 1. Our data indicated that the definition of subgroup has a significant effect on the outcome. However, it exhibited two common features with no subgroups and two subgroups; *CYP2*, *GADPH*, and *Tub1* were always classified among the top three positions, meaning that they have higher expression stabilities, and 18S was the worst reference gene. *CYP2* and *Tua5* were the most stable references in TRV-infected fruits, and *CYP2* and *UBQ10* were the most stable genes among different genotypes. *18S* and *TEF2* had the lowest stability under all experimental conditions. *18S*, *GADPH*, and *TEF2* were always included as unstable reference genes, showing an unstable expression profile.

#### 3.3.3. BestKeeper Analysis

The expression stability of the 10 reference genes was further analyzed using BestKeeper, and the R value was also calculated for assessing the stability. The higher the R value, the more it indicated stable expression of the reference gene (Table 2). Initial analysis of the data using CV values (coefficient of variation) and SD values (standard deviation) for 10 reference genes demonstrated that SD values of all reference genes were less than 1, except for six genes (*CYP2*, *Actin*, *Tub1*, *Tua5*, *TEF2,* and *GADPH*) in TRV-infected fruits, indicating that most were unstable expression under TRV infection in peach fruit. The most stable expressed genes were *CYP2* for 0.954, *Tua5* for 0.954 in all samples; *CYP2* for 0.999 and *Actin* for 0.991 under TRV-infected conditions; *Tua5* for 0.934 and *CYP2* for 0.918 in different genotypes. In addition, *18S* was the least stable gene with the highest stability in all samples, while *GADPH* and *PLA2* were the most unstable reference genes in TRV-infected fruits and different genotypes, respectively. It can be seen that *CYP2* expression is relatively stable and has been in the top two positions in all experimental conditions. The most unstable is *18S*, which has been in the last two places.

#### 3.3.4. RefFinder Analysis

For the final ranking of the reference genes, a further comprehensive stability analysis was performed on all 10 reference genes according to RefFinder. RefFinder considers the results from four different algorithms to provide comprehensive stable values of reference genes. The recommended comprehensive ranking was: *CYP2* and *Tub1* for total samples; *CYP2* and *Tua5* for TRV-infected fruits; *CYP2* and *Tub1* for different genotypes (Table 3). Furthermore, *TEF2* always included unstable reference genes under all experimental conditions.

### 3.4. Validation of Reference Genes

**Figure 4:**
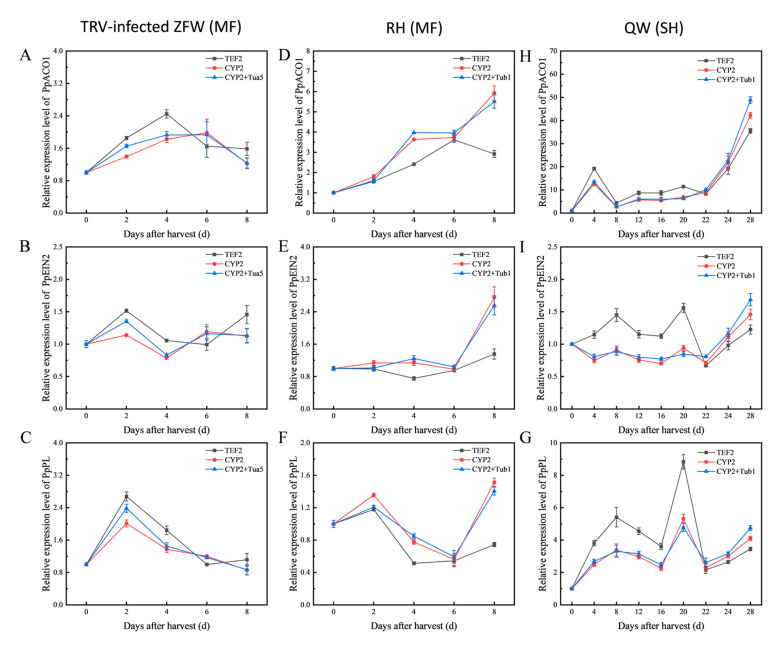
Relative expression levels of *PpACO1*, *PpEIN2*, and *PpPL* during peach fruit storage. The expression pattern was normalized by the best combination of reference gene, the most stable reference gene alone and the least stable reference gene. (**A**–**C**) gene expression levels in TRV-infected ‘Zao Feng Wang’ (ZFW) fruits during storage, (**D**–**F**) gene expression levels in ‘Rui Hong’ (RH) during storage, and (**H**–**G**) gene expression levels in ‘Qin Wang’ (QW) during storage. MF: melting flesh; SH: stony-hard. Data shown the means with standard error (*n* = 3).

To evaluate the stability of selected reference genes, the expression pattern of *PpACO1*, *PpEIN2,* and *PpPL* was evaluated during peach fruit ripening with the best combination of reference genes (*CYP2* with *Tua5* for TRV-infected fruits, *CYP2* with *Tub1* for different genotypes), the most stable genes (*CYP2*) and unstable genes (*TEF2*) for normalization (Figure 4). 

In terms of the TRV-injected fruits, it can be found that the expression levels of *PpACO1*, *PpEIN2,* and *PpPL* when normalized in combination with the stable reference gene and the best reference gene alone were consistent, but their expression patterns were partially similar to those of the others when normalized using the unstable reference gene *TEF2*. The expression level of *PpACO1* that was normalized by *CYP2* alone and the combination of *CYP2* and *Tua5*, gradually increased and reached its peak at 6 DAH (days after harvest), then decreased, whereas its expression level with *TEF2* as reference gene reached its peak at 4 DAH (Figure 4A).

During melting flesh peach fruit storage of ‘RH’, when the relative expression changes of *PpEIN2* were normalized by the combination of the stable reference gene and the best reference gene alone, the expression level of PpEIN2 remained essentially unchanged. However, when the most unstable reference gene TEF2 was used, the expression of PpEIN2 decreased from 2 DAH to 6 DAH, a significant change compared with the previous two results (Figure 4E). In the stony hard flesh peach fruit of ‘QW’, no differences were observed with different reference genes, indicating that the reference gene has different stability in different varieties.

## 4. Discussion

The reference gene should show minimal change and appropriate expression abundance in different tissues and experimental conditions [7,38]. There is a literature for screening reference genes in plants, such as peach [11], tomato [39,40], *Arabidopsis* [41], potato [42], soybean [43,44], banana [45], and longan [16], finding that the expression level of the internal reference is also unstable under every experimental condition. Therefore, the selection of internal reference genes under specific experimental conditions is particularly necessary. In this study, we aim to select reference genes that could be stably expressed as internal controls for RT-qPCR studies of gene expression in peach fruit with different genotypes and TRV-infected fruits.

We analyzed the expression profiles of 10 reference genes. The reference gene should meet the appropriate Ct value range. In this study, the average Ct values of the 10 reference genes showed that *18S* had the narrowest Ct range, while *TEF2* had the widest (Figure 1). Taking *18S* as an example, a small Ct value reflects a large expression accumulation, indicating that the expression abundance of *18S* is particularly high. The narrow Ct value range can reflect the stability to a certain extent, indicating that the *18S* has the best stability (Figure 1). However, in this experiment, *18S* was not the best choice for the internal reference gene. The reason is as follows: because of its abundance, the *18S* is recommended only for target genes that are highly transcribed. When the target gene expression level is low, the *18S* is not suitable as a reference gene for accurate subtraction of baseline values in RT-qPCR data analysis [9,46]. Furthermore, *18S* was the most unstable reference gene in all the samples from geNorm and RefFinder analysis (Figure 2; Table 3). The stability of *18S* in peach was also found to be poor [9,32]. It is precisely for this reason that the *18S* failed to replace the use of other housekeeping genes. This demonstrated that it needs to combine the expression levels of the reference genes with stability analysis.

The stability of the gene expression was confirmed separately using geNorm, NormFinder, BestKeeper, and RefFinder for ranking reference genes. The geNorm program calculates the M value of the stability of each reference gene to screen out the more stable reference genes [34]. NormFinder software obtains expression stability values based on the intra- and inter-group variation coefficient of each reference gene between samples [35]. BestKeeper comprehensive analysis of the stability of reference genes based on the SD, CV and R of Ct values of each reference gene under various experimental conditions [36]. Furthermore, RefFinder considers the results from four different algorithms, including geNorm, NormFinder, BestKeeper, and ΔCt method, to provide comprehensive stable values of reference genes [37]. We use four most popular software packages to analyze the stability of 10 reference genes. Among TRV-infected fruits, the geNorm, NormFinder, BestKeeper, and RefFinder analyses showed almost identical top five stable reference genes, although with a slightly change in rank order. Moreover, the ranking orders of reference genes under different genotypes by the four programs are different, which is expected because the algorithms of the software are different.

For RefFinder analysis, *CYP2*, *Tua5*, and *Actin* were ranked as the top three in TRV-infected fruits, followed by *CYP2*, *Tub1*, and *UBQ10* in different genotypes. In addition, *GADPH*, *TEF2,* and *PLA2* were ranked as the last three in TRV-infected fruits, with *GADPH*, *TEF2,* and *18S* in different genotypes (Table 3). Although most authors used only a single reference gene as a standardized internal control, it has been suggested that RT-qPCR studies using two or more reference genes may contribute to more reliable results [12,47,48,49]. According to pairwise variation, two reference genes were required for gene expression level normalization in this study since the V2/3 was lower than 0.15 (Figure 3). Therefore, the best reference gene combinations were *CYP2* with *Tua5* for TRV-infected fruits and *CYP2* with *Tub1* for different genotypes. To further confirm the validated reference genes in RT-qPCR, the combination of the most stable reference gene, the best reference gene alone, and the least stable reference gene were used for normalization of *PpACO1*, *PpEIN2*, and *PpPL*. The result shows that normalization by the unstable reference gene leads to misinterpretation of the expression pattern of *PpACO1*, *PpEIN2*, and *PpPL* (Figure 4). Therefore, screening for stable reference genes is highly necessary for the correct processing of RT-qPCR data.

In this study, *CYP2*, ranked in the top two by geNorm, NormFinder, and Bestkeeper programs, proved to be the best reference gene for all experimental conditions (Figure 2; Table 1 and Table 2). *CYP2* was the best choice for standardization in pulp samples at different storage temperatures and fruit development stages in a pre-study of peach [11], similar to our result. In geNorm analysis, *CYP* has maintained the top six positions of 19 genes throughout the developmental stages and postharvest ripening of peel and pulp, indicating *CYP* has better stability during banana fruit development and maturation [45]. *CYP2* presents stable expression in different developmental stages and after 2,4-D treatment of longan [16]. Furthermore, in cucumbers, *CYP* was best reference gene under cold and heat treatment [50]. This suggests that the stability of *CYP2* expression is reliable across species.

It has been reported in peach that *TEF2* was found to be the most stable and reliable reference gene, suggesting the use of *TEF2* in combination with *RPII* for different tissues, different storage times and different genotypes [9]. However, in this study, *TEF2* was the most unstable reference gene in different genotypes by RefFinder analysis (Table 3), and the top three reference genes with high variability among all experimental conditions by Normfinder analysis (Table 1). The expression level of *PpACO1* normalized by *TEF2* displayed differences in the expression level with the combination of the most stable reference gene and the most stable reference gene alone in ‘RH’ during ripening (Figure 4D). These results show that *TEF2* is not the best normalization factor for different genotypes. It is possible that the different varieties of peach and experimental conditions have led to the discrepancy between the two assays [13]. The poor stability of *TEF2* was also pointed out in peach and other species. *TEF2* exhibited a middle-rank order for flesh at different storage temperatures and different tissues, and did not perform as well as other stable genes as a normalization factor in peach [11]. The least appropriate reference gene was *TEF2* for seed development and the entire growth cycle in *Plukenetia volubilis* [51].

## 5. Conclusions

To our knowledge, this is the first report on the validation of a set of reference genes in TRV-infected peach fruits for the standardization of gene expression analysis using RT-qPCR. Our results suggest that it need to select the expression stability of reference genes under specific experimental conditions. For TRV-infected fruits, *CYP2* with *Tua5* is best gene combination for gene normalization. For different genotypes, *CYP2* with *Tub1* is one of the best gene combinations. Our results provide a foundation for the further use of RT-qPCR in the analysis of gene expression in peach.

## Figures and Tables

**Table 1:** Ranking of variation value for ten reference genes calculated by Normfinder.

Rank	All Samples				TRV-Infected Fruits		Different Genotypes	
	No Subgroups		2 Subgroups					
1	CYP2	0.192	RP II	0.110	CYP2	0.125	CYP2	0.222
2	RP II	0.381	CYP2	0.133	Tua5	0.211	UBQ10	0.404
3	Tub1	0.407	Tub1	0.218	RP II	0.226	RP II	0.426
4	UBQ10	0.417	Tua5	0.227	Actin	0.252	Tub1	0.432
5	Tua5	0.437	UBQ10	0.240	Tub1	0.267	Tua5	0.470
6	Actin	0.478	PLA2	0.291	UBQ10	0.433	Actin	0.476
7	GADPH	0.625	Actin	0.312	PLA2	0.471	GADPH	0.544
8	PLA2	0.691	TEF2	0.343	GADPH	0.775	PLA2	0.739
9	TEF2	0.794	GADPH	0.350	TEF2	0.815	TEF2	0.785
10	18S	0.980	18S	0.444	18S	0.816	18S	0.969

**Table 2:** The R values of ten reference genes were calculated by BestKepper.

Rank	All Samples			TRV-Infected Fruits			Different Genotypes		
		CV ± SD	R		CV ± SD	R		CV ± SD	R
1	CYP2	3.10 ± 0.81	0.954	CYP2	3.97 ± 1.01	0.999	Tua5	3.24 ± 0.81	0.934
2	Tua5	4.02 ± 0.99	0.954	Actin	5.88 ± 1.20	0.991	CYP2	2.40 ± 0.63	0.918
3	TEF2	5.18 ± 1.16	0.928	Tub1	5.37 ± 1.20	0.989	Actin	4.05 ± 0.84	0.907
4	RP II	3.23 ± 0.79	0.916	Tua5	4.80 ± 1.15	0.984	RP II	2.71 ± 0.67	0.881
5	Actin	4.66 ± 0.97	0.915	TEF2	7.64 ± 1.65	0.978	TEF2	4.13 ± 0.94	0.867
6	Tub1	3.66 ± 0.84	0.896	RP II	3.99 ± 0.95	0.971	UBQ10	2.64 ± 0.59	0.835
7	UBQ10	3.51 ± 0.78	0.891	UBQ10	3.43 ± 0.73	0.969	GADPH	3.39 ± 0.73	0.815
8	GADPH	4.55 ± 0.96	0.848	PLA2	3.27 ± 0.90	0.966	Tub1	2.83 ± 0.65	0.803
9	PLA2	2.62 ± 0.74	0.741	18S	5.72 ± 0.59	0.957	18S	5.75 ± 0.60	0.437
10	18S	5.87 ± 0.61	0.554	GADPH	5.40 ± 1.09	0.145	PLA2	1.66 ± 0.47	0.435

**Table 3:** Comprehensive ranking of ten reference genes were calculated by RefFinder.

Rank	All Samples		TRV-Infected Fruits		Different Genotypes	
1	CYP2	1.50	CYP2	2.11	CYP2	1.41
2	Tub1	2.45	Tua5	3.03	Tub1	2.51
3	RPII	3.13	Actin	3.22	UBQ10	2.63
4	UBQ10	4.12	Tub1	3.56	RPII	4.61
5	Actin	5.42	RPII	4.16	PLA2	4.76
6	18S	5.62	UBQ10	4.56	Actin	5.05
7	PLA2	5.66	18S	5.62	Tua5	6.16
8	Tua5	5.79	PLA2	5.66	GADPH	7.00
9	GADPH	7.00	GADPH	7.90	18S	7.40
10	TEF2	9.24	TEF2	8.71	TEF2	9.24

## Data Availability

Not applicable.

## Supplementary Materials

The following supporting information can be downloaded at: https://www.mdpi.com/article/10.3390/genes13010160/s1, Figure S1: The amplification sizes and specificity of primers of ten reference genes. M, DL 2000 maker. Figure S2: Melting curve of ten reference genes by RT-qPCR. A single peak indicated the specificity of primers. Table S1: Sample sets in this study. Table S2: Delineations of ten candidate reference genes. Table S3: The information of the primers used for qRT-PCR validation.
