# Supplementary material for: Selection and Validation of Reliable Reference Genes for Gene Expression Studies in Different Genotypes and TRV-Infected Fruits of Peach (Prunus persica L. Batsch) during Ripening
Source: Genes (Basel). 2022 Jan 17;13(1):160. doi: 10.3390/genes13010160 (PMC8775616; doi:10.3390/genes13010160)
Supplement: Supplementary file 1 [file genes-13-00160-s001.zip › genes-1532245-Supplementary.pdf]

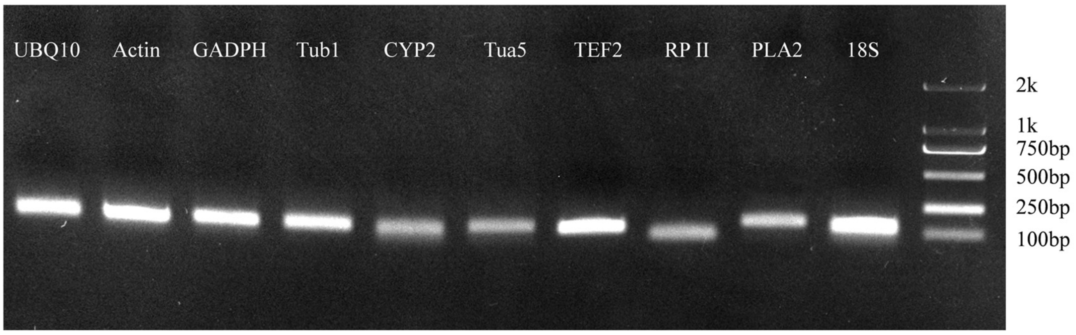

Figure S1. The amplification sizes and specificity of primers of ten reference genes. Amplified fragments of 10 reference genes exhibited by 1% agarose gel electrophoresis. M, DL 2000 maker.

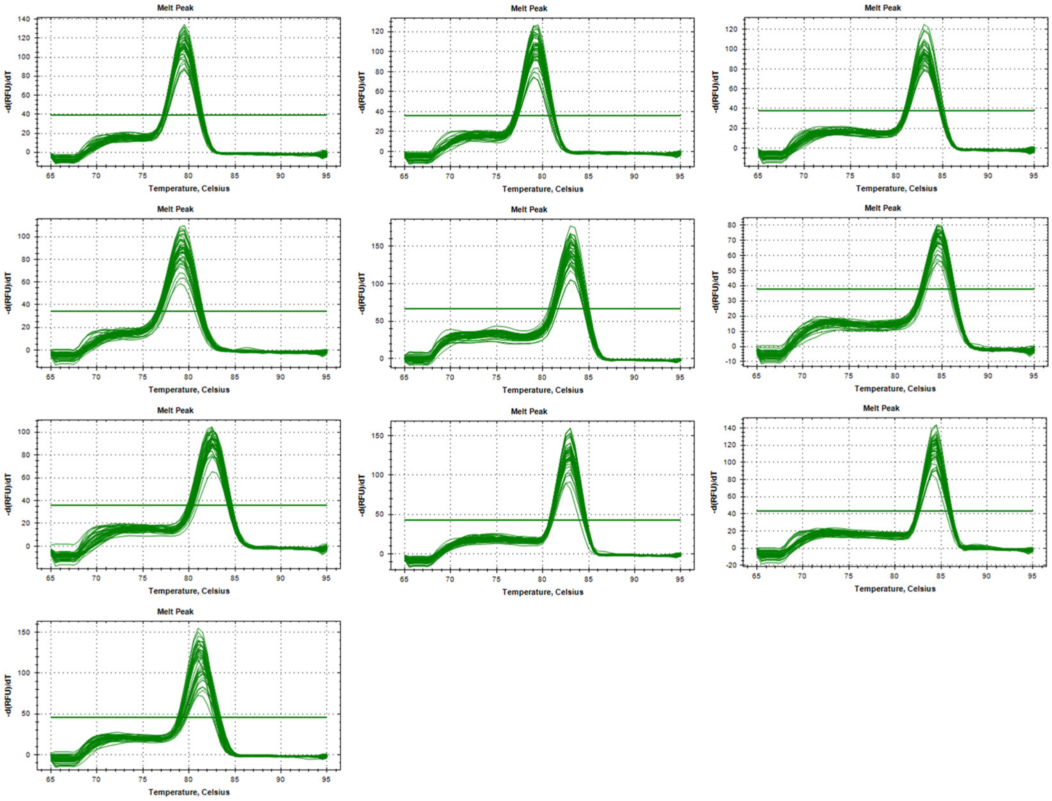

Figure S2. Melting curve of ten reference genes by RT-qPCR. A single peak indicated the specificity of primers.

Table S1. Sample sets in this study.

Experimental samples sets	Variety	Sampling types
TRV-infected fruits	Rui Hong	0d, 2d, 4d, 6d
	Zao Feng Wang	0d, 2d, 4d, 6d, 8d
different genotypes	Rui Hong	0d, 2d, 4d, 6d, 8d
	Zao Feng Wang	0d, 2d, 4d, 6d, 8d
	Babygold 5	0d, 2d, 4d, 6d, 8d, 10d, 12d, 14d,16d, 18d
	Qin Wang	0d, 4d, 8d, 12d, 16d, 20d, 22d, 24d, 28d

Table S2. Delineations of ten candidate reference genes.

Symbol	Gene name	Primer sequence(5'-3')(Forwad/Reverse)	Size(bp)	Efficiency(%)	R^2	Reference
18S	18S ribosomal RNA	TAGTTGGTGGAGCGATTTGTCTG/<br>CTAAGCGGCATAGTCCCTCTAAG	114	1.8	0.99	Tong et al., 2009, Xu et al., 2008, Kou et al., 2016
Actin	Actin protein	GATTCCGGTGCCCAGAAGT/<br>CCAGCAGCTTCCATTCCAA	144	1.8	0.99	Leida et al., 2010, Tatsuki et al., 2013
CYP2	Cyclophilin 2	ACTCCAAAGCGTGTTAGAAAAGG/<br>GTCTCTTCCACCATAACGATAGG	120	1.7	0.99	Tong et al., 2009, Kou et al., 2016
TEF2	Translation enlongation factor 2	GGTGTGACGATGAAGAGTGATG/<br>TGAAGGAGAGGGAAGGTGAAAG	129	1.8	0.99	Tong et al., 2009, Kou et al., 2016
GAPDH	Glyceraldehyde-3- phosphate dehydrogenase	ATTTGGAATCGTTGAGGGTCTTATG/<br>AATGATGTTGAAGGAAGCAGCAC	121	1.8	0.99	Tong et al., 2009, Kou et al., 2016

PLA2	Phospholipase A2 beta	TCGCCGTCGTTATCTTCTCC/ TACCGAATCCCAACAGAATTACAG	115	1.7	0.99	Tong et al., 2009, Kou et al., 2016
RP II	RNA polymerase subunit	TGAAGCATACACCTATGATGATGAAG/ CTTTGACAGCACCAGTAGATTCC	128	1.8	0.99	Tong et al., 2009, Kou et al., 2016
Tua5	Tublin alpha-5	TTCTCTCTACTCATTCCCTCCTTG/ GATTGGTGTATGTTGGTCTCTCG	117	1.8	0.99	Tong et al., 2009，Li et al., 2009 , Kou et al., 2016
Tub1	Tublin beta-1	CCGAGAATTGTGACTGCCTTCAAG/ AGCATCATCCTGTCTGGGTATTCC	124	1.8	0.99	Tong et al., 2009, Kou et al., 2016
UBQ10	Ubiquitin 10	AAGGCTAAGATCCAAGACAAAGAG/ CCACGAAGACGAAGCACTAAG	146	1.8	0.99	Tong et al., 2009, Kou et al., 2016

Table S3. The information of the primers used for qRT-PCR validation.

Symbol	Gene name	Gene number	Primer sequence(5'-3')(Forwad/Reverse)
PpACO1	1-aminocyclopropane-1-carboxylate oxidase	ppa008791m	CCCCCATGCGCCACTCCA/ CATCACTGCCAGGGTTGTAAAAG
PpEIN2	ethylene insensitive 2	ppa000305m	ACTACCAGCCAGCCACAATACA/ GCACCCAATGAAGAAGCGGATT
PpPL	pectate lyase	ppa006392m	ACAGGCAGAGGCTGGCAGATT/ GGAGGACCGCACCATAGCATTC
